# *In vitro* and *in vivo* properties of the bovine antimicrobial peptide, Bactenecin 5

**DOI:** 10.1371/journal.pone.0210508

**Published:** 2019-01-09

**Authors:** R. L. Price, L. Bugeon, S. Mostowy, C. Makendi, B. W. Wren, H. D. Williams, S. J. Willcocks

**Affiliations:** 1 Department of Life Sciences, Imperial College London, London, United Kingdom; 2 Department of Medicine, Imperial College London, United Kingdom; 3 Department of Pathogen Molecular Biology, The London School of Hygiene and Tropical Medicine, London, United Kingdom; Emory University School of Medicine, UNITED STATES

## Abstract

Antimicrobial peptides (AMP), part of the innate immune system, are well studied for their ability to kill pathogenic microorganisms. However, many also possess important immunomodulatory effects, and this area has potential for the development of novel therapies to supplement traditional methods such as the use of antibiotics. Here, we characterise the microbicidal and immunomodulatory potential of the proline-rich bovine AMP, Bactenecin 5 (Bac5). We demonstrate broad antimicrobial activity, including against some mycobacterial species, which are important pathogens of fish, cattle and humans. Bac5 is able to activate macrophage-like THP-1 cells and can synergistically trigger the upregulation of *tnf-α* when co-stimulated with *M*. *marinum*. Furthermore, Bac5 sensitises A549 epithelial cells to stimulation with TNF-α. For the first time, we characterise the activity of Bac5 *in vivo*, and show it to be a potent chemokine for macrophages in the zebrafish (*Danio rerio*) embryo model of infection. Bac5 also supports the early recruitment of neutrophils in the presence of *M*. *marinum*. In the absence of host adaptive immunity, exogenous injected Bac5 is able to slow, although not prevent, infection of zebrafish with *M*. *marinum*.

## Introduction

The rising incidence of antimicrobial resistance and decline in major commercial investment into new antimicrobial development is driving the innovative use of alternative compounds and novel strategies to combat infection. Antimicrobial peptides (AMP) are a conserved but diverse group of innate immune effectors that have been well studied for their ability to kill bacteria, fungi and viruses (reviewed by Radek *et al* [1]). They are usually small, typically below fifty amino-acids in length, tend to be cationic and hydrophobic, and can have an array of secondary structures and modifications. The sheer diversity of possible sequence variations lends them to the rational design of novel AMP derivatives, which is a promising avenue of research [2–6]. The study of naturally occurring AMP can be used to identify those that carry properties that may be desirable in new therapeutics, as well as elucidating their natural role during infection.

While some AMP act through direct lysis of the bacterial cell membrane, others target intracellular sites, including heat-shock proteins and ribosomal subunits (reviewed by Mishra *et al* [7]). Furthermore, some have been reported to contribute to wound healing [8] and angiogenesis [9]. The only human AMP belonging to the cathelicidin-family is LL37; which, in addition to human beta defensin (HBD)3, has been described as chemotactic for neutrophils, T-cells [10], macrophages [11], immature dendritic cells [10] and mast cells [11]. It is these non-bactericidal properties of AMP that are garnering interest for their potential application as immunoregulators to treat disease. An attractive aspect of this approach is that since the AMP does not depend on direct microbicidal activity, rather supporting the host immune response to eradicate infection, it may confer less selective pressure to evolve resistance. Immunomodulatory AMPs have already entered clinical trials for the treatment of acne, rosacea and malaria [12].

Bactenecins (Bac) comprise a family of cyclic peptides released by activated bovine neutrophils upon degranulation [13]. Bac5, also known as bovine cathelicidin-2, is unusual in that it is particularly arginine-proline rich, a feature normally associated with insect and crustacean AMPs, and otherwise so far only identified in ruminants.

Since it lacks any cysteine residues, Bac5 cannot form disulphide bridges and exists in a linear conformation [14]. Linearised forms of other bactenecins are reportedly less active against Gram-negative species, but more active against Gram-positive species, compared with their cyclised form [15]. Bac5 is reportedly non-lytic [16] and can be internalised by some bacteria via a transport-mediated mechanism to reach intracellular targets [17]. However, there are several reports that Bac5 can also fuse with and alter the morphology of bacterial membranes, and this correlates with microbicidal activity [18, 19]. Bac5 demonstrates species-restricted bactericidal activity, particularly against Gram-negative bacteria including *Escherichia coli*, *Burkholderia pseudomallei* [20] and *Klebsiella sp*. [21], but retains some limited activity against certain Gram-positive species: it can kill *Staphylococcus epidermidis*, for example, but not *S*. *aureus*. Meanwhile it has also been shown to be able to kill *Candida albicans* [22]. To our knowledge, the activity of bovine Bac5 against mycobacteria has not been investigated.

Although synthesised during myelopoiesis [23], Bac5 is also strongly upregulated upon intramammary challenge with *Streptococcus uberis* [24] and *E*. *coli*, although in goats it appears to be constitutively expressed in healthy somatic milk cells [25]. Interestingly, despite its transcriptional upregulation in this tissue, the peptide itself has dramatically reduced minimum inhibitory concentration (MIC) in bovine serum and milk [26]. Still, the pro-form of the peptide may retain chemotactic properties that are still of benefit to the host, similar to the related peptide, Bac7 [27]. Proteolytic cleavage of pro-Bac5 to the mature Bac5 form is thought to occur rapidly, within five minutes [13, 28], while mRNA expression of Bac5 appears to peak at around 20 hours post-stimulation of neutrophils with *E*. *coli* as stores of the pro-peptide are replenished [29]. These findings point to an important sentinel role for Bac5 in peripheral tissue in preventing infection. However, the non-bactericidal effects of Bac5, particularly with regards to important host cell types involved in innate immunity, as well as in the context of mycobacterial infection have not been well studied.

Proline-rich peptides, such as those isolated from bovine colostrum, have been shown to counteract the Th2-dominated allergic inflammatory response and promote a Th1-associated outcome, for example by the induction of IFN-γ and TNF-α in human leukocytes and whole blood cultures [30]. It is believed that this type of immune response may be important in protection against progressive mycobacterial infection [31]. Immunomodulation by other bovine AMP has been reported, with indolicidin shown to induce CXCL8; and Bac7, structurally similar to Bac5, potentially able induce DNA synthesis and proliferation of fibroblasts [28]. Some promising research has demonstrated that some AMP can neutralise lipopolysaccharide and reduce TLR4-mediated inflammation, offering a potential new treatment for sepsis through detoxification [32–38]. Certain modern synthetic peptides derived from the native Bac2 sequence are also able to upregulate expression of potent chemokines such as MCP-1 [39].

In this study, we sought to investigate the bactericidal and immunomodulatory properties of Bac5 *in vitro* and *in vivo*, in the presence and absence of mycobacterial infection. We use the zebrafish (*Danio rerio*) embryo infection model along with its natural pathogen, *M*. *marinum* [40, 41]. This system confers several advantages: the zebrafish embryos are transparent and therefore amenable to fluorescence imaging of bacteria and host cell types [42]; they do not encode proline-rich AMP, enabling the study of Bac5 in isolation; and finally, they lack a developed adaptive immune response (reviewed by Trede *et al* [43]), enabling us to characterise the capabilities and limitations of Bac5 in a purely innate context.

## Results

### Bac5 Has species-selective antibacterial activity

**Fig 1:**
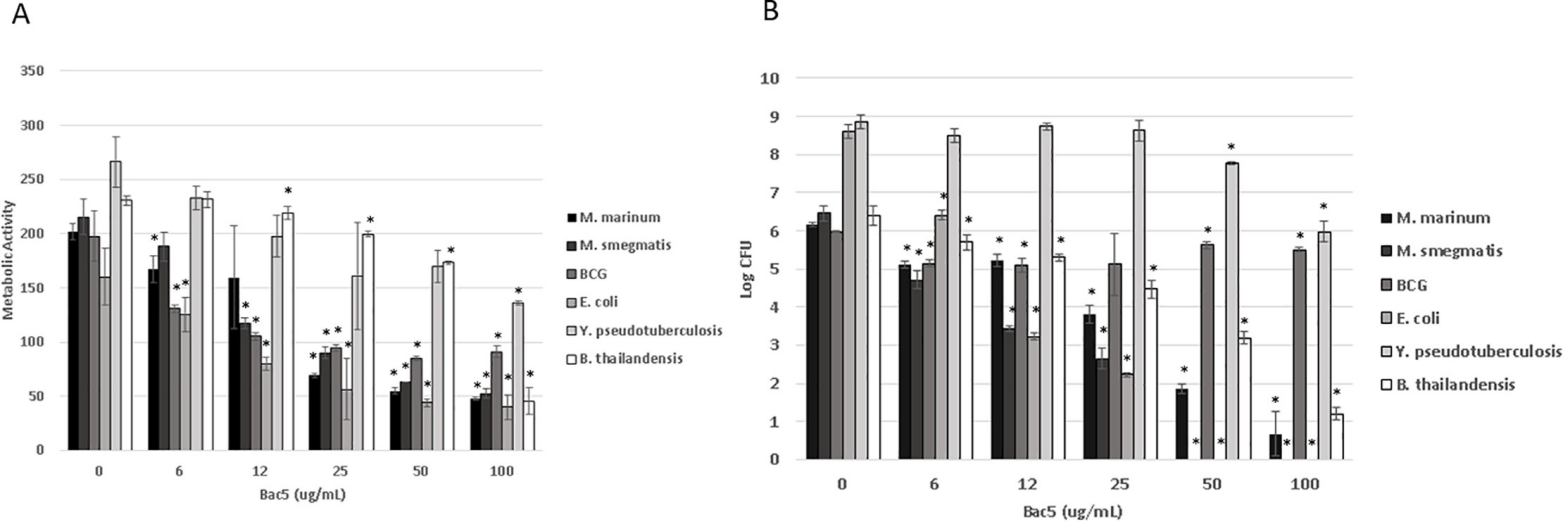
Antibacterial effect of Bac5 against gram-negative and Acid-Fast bacilli. The active fragment of Bac5 was synthesised to high purity and incubated at different concentrations with mid-log phase bacteria of different species in a sterile solution of RPMI:Water (1:4, v:v). Metabolic activity (**A**) was calculated using the colorimetric Alamar Blue assay, while bactericidal activity was recorded by colony forming unit (CFU) assay (**B**). All data were collected at 24 hours, except for *M*. *bovis* BCG, where metabolic activity was recorded after 72 hours. Stars denote statistical (p< 0.01) significance from the untreated (zero) control for each species as calculated using Student’s T-test.

The active portion of the Bac5 amino acid sequence was synthesised, purified and tested for activity against different Gram-negative and Acid-Fast species of bacteria. The peptide demonstrated significant, concentration-dependent ability to restrict metabolic activity in all bacteria species tested (**Fig 1A**). Bac5 was variably microbicidal against all species, mostly correlating well with the metabolic assay, although *Y*. *pseudotuberculosis* and *M*. *bovis* BCG both demonstrated greater resistance to the peptide, and required higher concentration of Bac5 to significantly reduce CFU (**Fig 1B**).

### Bac5 modulates chemokine and cytokine transcriptional activity in response to *M*. *marinum* and *tnf*-*α* challenge *in vitro*

**Fig 2:**
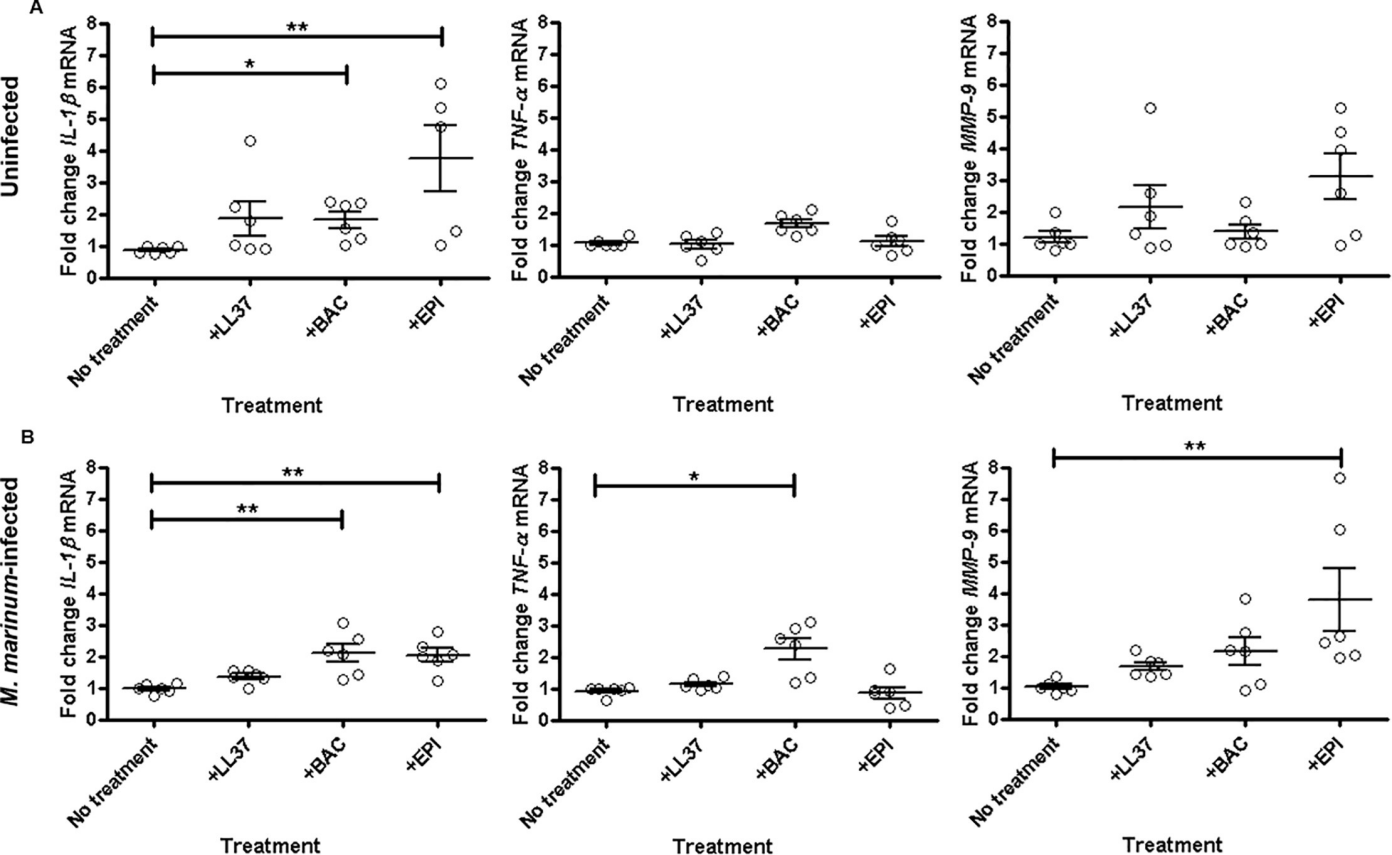
Effect of Bac5 on THP-1 cell cytokine transcription during infection. Fold change of *IL-1β*, *TNF-α* and *MMP-9* mRNA levels in uninfected **(A)** and *M*. *marinum-*infected **(B)** resting THP-1 cells following incubation with 15 ng/μl AMPs for 24 h. Fold changes of mRNA levels were assessed by qRT-PCR, normalised to 18S and expressed relative to one control (no treatment) sample. Data pooled from two independent experiments is shown. Each data point represents two treatment wells (n = 12). Error bars represent S.E.M. Kruskal-Wallis with Dunn’s post-test. **p<0.01, *p<0.05.

We characterised the immunomodulatory effect of Bac5 *in vitro* by looking directly at its effect on key cell types and cytokines associated with mycobacterial infection. We studied the effect of Bac5 on matured human THP-1 macrophage-like cells, and found that synthetic Bac5 and epinecidin induced the transcription of *IL-1β* both in the presence and absence of live *M*. *marinum* in THP-1 cells (**Fig 2A & 2B**). Interestingly, Bac5 was the only peptide tested to significantly upregulate *tnf-α*, but only in synergy with *M*. *marinum*. Epinecidin significantly upregulated both *mmp-9* and *IL-1β*, particularly in the presence of the bacteria. Collectively, these results show that Bac5 is able to activate macrophage-like cells *in vitro* in the absence of additional host cell types or cytokines.

We looked for the same cytokine responses *in vivo* using the zebrafish embryo model, following infection with *M*. *marinum* and treatment with a single injection of 10 ng Bac5 in the hindbrain ventricle (HBV). Whilst *M*. *marinum* induced *il-1β*, *mmp-9* and *cxcl-c1c* after 96 hours, Bac5 did not significantly affect this outcome (**S1 Fig**), in contrast to our *in vitro* findings. To establish whether injection of Bac5 at later stages post-infection influenced the cytokine profile, we allowed the infection to proceed to either 72 or 90 hpi before treating with Bac5, and again found no significant difference from the untreated control (**S2 Fig**).

To examine whether endogenously-produced Bac5 could also modulate chemokine and cytokine responses by host cells *in vitro*, we transformed the adherent human epithelial cell line, A549 to stably express Bac5. We confirmed *Bac5* expression by A549 cell lines by both transcriptional analysis and also by phenotypic assay, confirming that, as with the synthetic version of the peptide, A549 cells expressing Bac5 could inhibit bacterial metabolic activity. The finding that this only occurred in culture supernatant and not lysate indicated that the peptide was readily secreted (**S3A and S3B Fig**). We furthermore examined the effect of endogenously-produced Bac5 in response to stimulation with either live mycobacteria or to the inflammatory cytokine, TNF-α. Bac5 only weakly affected MMP-9 and CXCL8 secretion by epithelial cells when stimulated with mycobacteria, but significantly upregulated both cytokines in response to TNF-α stimulation in the absence of bacteria (**S3C and S3D Fig**).

### Bac5 induces rapid, sustained macrophage and transient neutrophil recruitment *in vivo*

**Fig 3:**
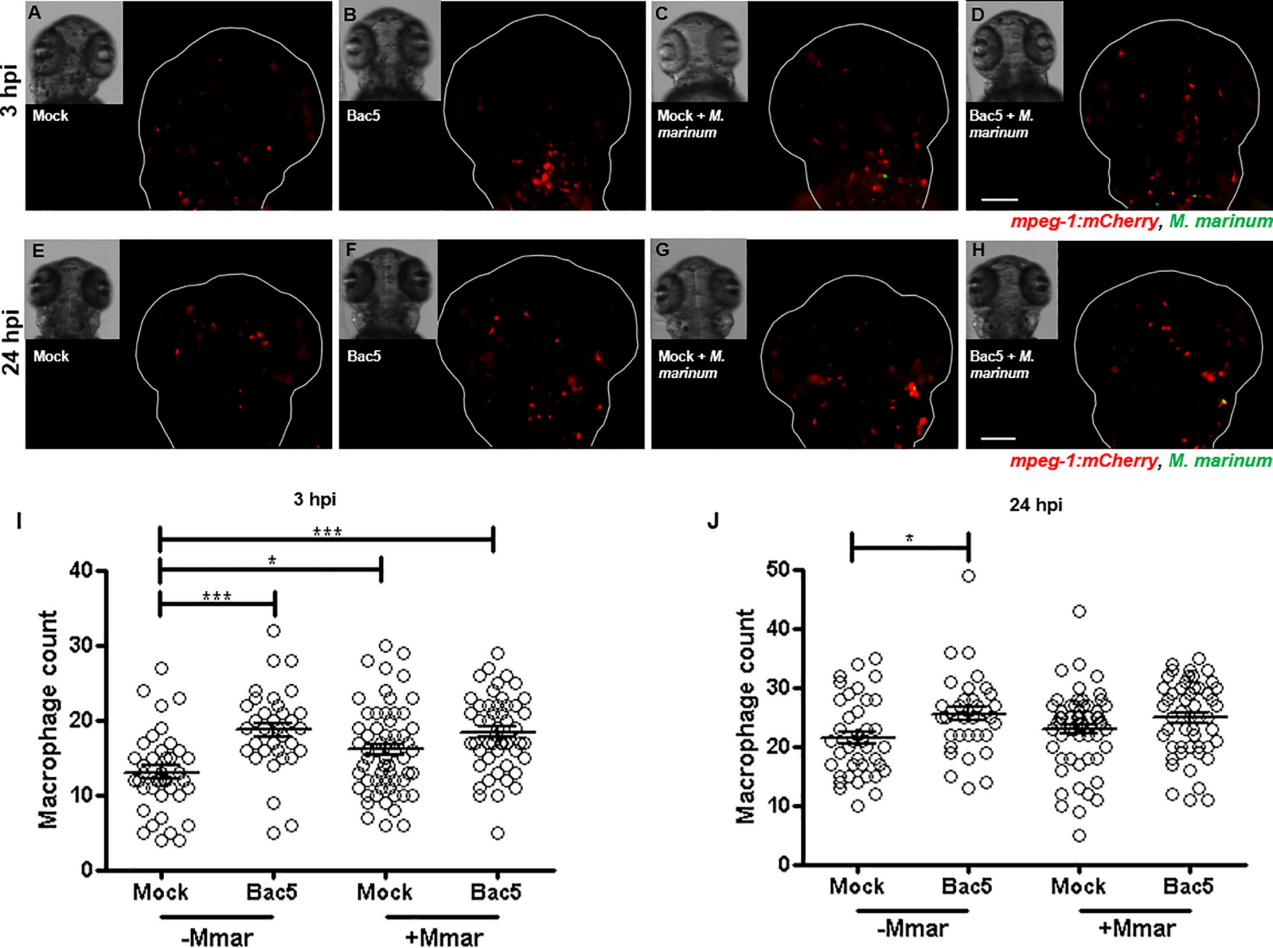
Injection of Bac5 to the hindbrain ventricle (HBV) increases macrophage recruitment. Two days post-fertilisation *Tg(mpeg-1*:*mCherry)* zebrafish embryos were injected into their HBV with Mock, BAC, Mock/*M*. *marinum* (Mmar) or BAC/Mmar. 10 ng Bac5 was injected for both Bac5 treatment groups and approximately 190 CFU Mmar expressing GFP for both infection groups. Z-stack images of the HBV region were acquired at 3 hours post-injection (hpi) and 24 hpi. **(A-H)** Representative fluorescence overlay images of fish at 3 hpi (A-D) and 24 hpi (E-H) from a single experiment are shown. Scale bar 100 μm. **(I-J)** Macrophages in the HBV region were quantified from fluorescence images of zebrafish taken at 3 hpi (I) and 24 hpi (J) using Icy Spot Detector plugin. Data pooled from three independent experiments is shown. Sample size (n): 40, 36, 61, 53. Error bars represent S.E.M. One-way ANOVA with Bonferroni’s post-test. *p<0.05, ***p<0.001.

Infection of the zebrafish embryo HBV with *M*. *marinum* induced the recruitment of macrophages to the site from 3–24 hpi (**Fig 3**). In the absence of bacteria, 10 ng Bac5 was also able to induce recruitment of macrophages, however co-injection of bacteria with peptide did not significantly enhance macrophage recruitment above the levels induced by bacteria alone. Beyond 24 hpi, macrophages aggregated into nascent granulomatous-like structures and were no longer discernible as discrete cells.

**Fig 4:**
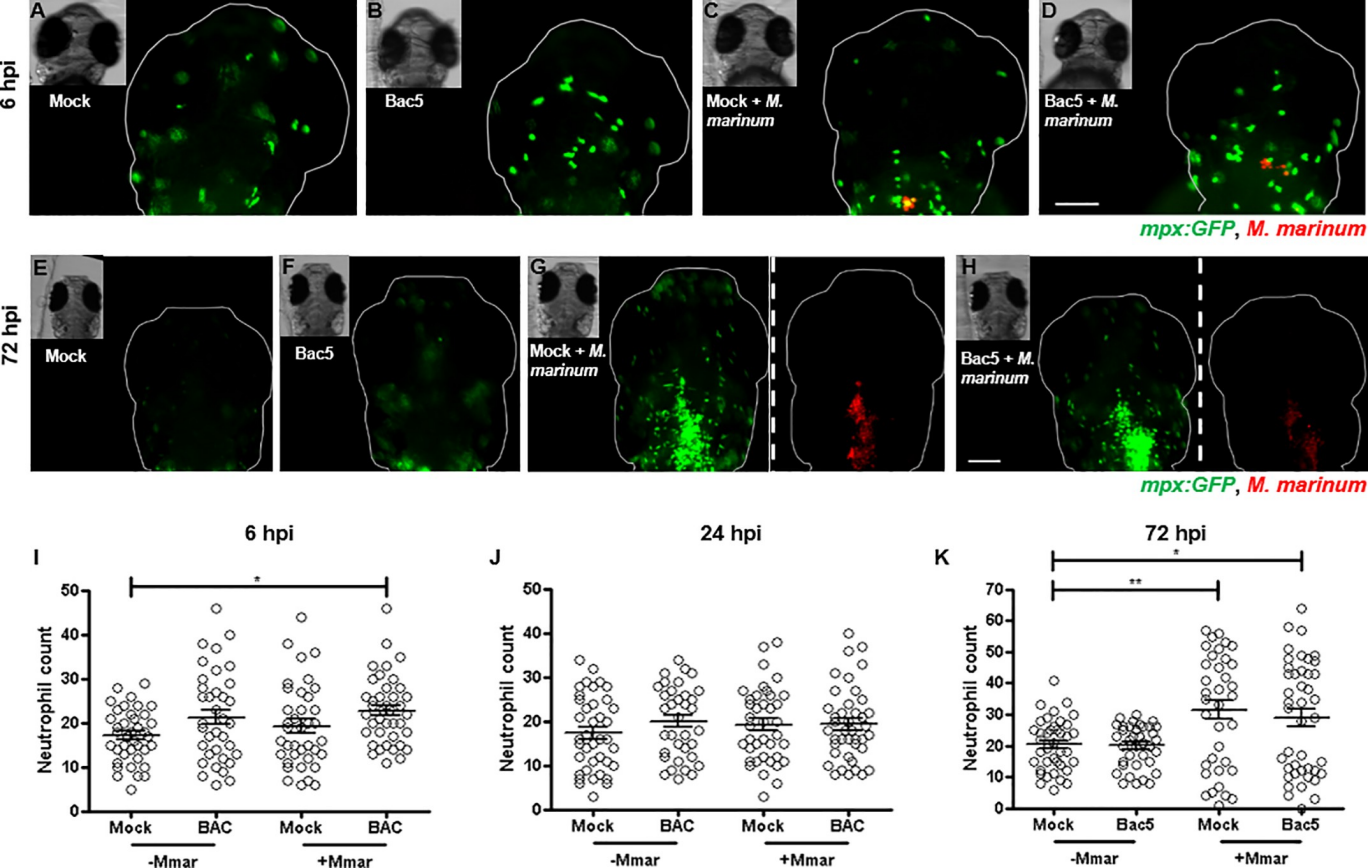
Injection of Bac5 to the HBV induces early but not sustained neutrophil recruitment. Two days post-fertilisation *casper Tg(mpx*:*GFP)* zebrafish embryos were injected into their HBV with Mock, Bac5, Mock/*M*. *marinum* (Mmar) or Bac5/Mmar. 10 ng BAC was injected for both BAC treatment groups and approximately 250 CFU Mmar expressing DsRed2 for both infection groups. *Z*-stack images of the HBV region were acquired at 6 hpi, 24 hpi and 72 hpi. **(A-D)** Representative fluorescence overlay images of fish from a single experiment are shown at 6 hpi. Scale bar 100 μm. **(E-H)** Representative fluorescence images of fish from the same single experiment are shown at 72 hpi. Images of the bacterial fluorescence channel are shown only for infected zebrafish groups, separated by white dashed line from the neutrophil channel images. Scale bar 100 μm. **(I-K)** Neutrophils in the HBV region were quantified from fluorescence images of zebrafish embryos using Icy Spot Detector plugin. From L to R: 6 hpi, 24 hpi and 72 hpi. Data was also analysed using Icy FPC protocol with the same outcome (S5 Fig). Sample size (n) = 39, 37, 38, 43. Data pooled from three independent experiments is shown. Error bars represent S.E.M. One-way ANOVA with Bonferroni’s post-test. *p<0.05, **p<0.01, ***p<0.001.

In contrast to macrophages, *M*. *marinum* did not induce neutrophil recruitment until 72 hpi. Interestingly, co-injection with Bac5 enabled the recruitment of neutrophils significantly earlier, at 6 hpi **(Fig 4**). Yet by 72 hpi, there was no discernible effect of Bac5 above the level of neutrophils recruited by *M*. *marinum* alone. Similarly, delayed injections of Bac5 at later time points (48 hpi and 96 hpi) showed that there was no significant difference in neutrophil count compared with *M*. *marinum* alone (**S4 Fig**).

### Co-injection of Bac5 with *M*. *marinum* slows the rate of bacterial infection

**Fig 5:**
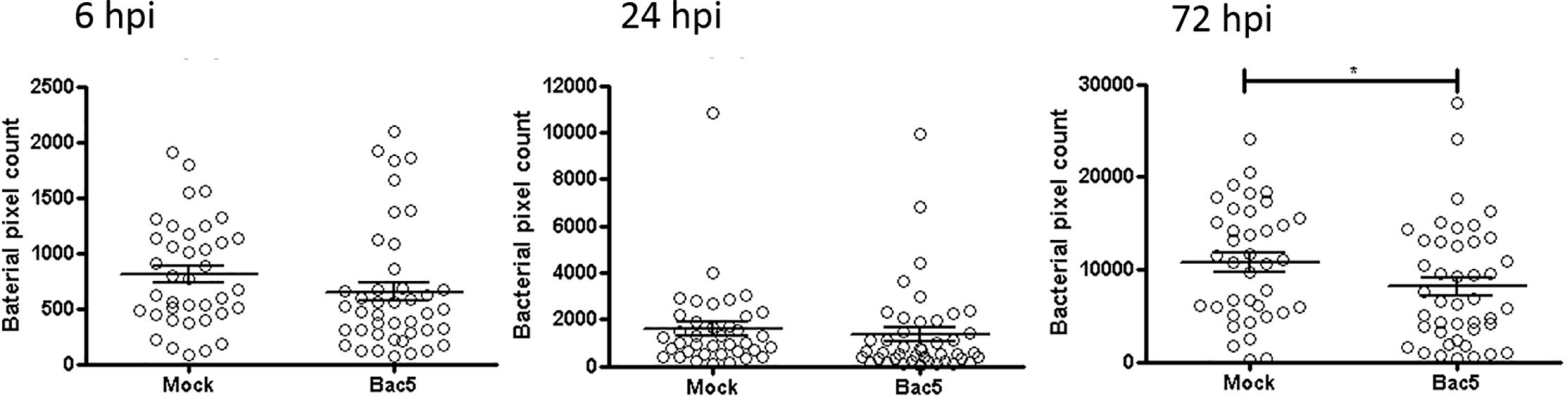
Exogenous injected Bac5 slows rate of infection with *m*. *marinum* in zebrafish embryos. Two days post-fertilisation *casper Tg(mpx*:*GFP)* zebrafish embryos were injected into their HBV with 10 ng Bac5 or mock control along with approximately 250 CFU Mmar expressing DsRed2 for both infection groups. Zebrafish embryos were live-imaged using a fluorescence stereomicroscope and z-stack images of the HBV region acquired at 6 hpi, 24 hpi and 72 hpi. Bacterial burden in the HBV region of *M*. *marinum*–infected zebrafish embryos was quantified from fluorescence images using Icy FPC protocol. Sample size (n): 38, 43. Data pooled from three independent experiments is shown. Error bars represent S.E.M. Mann-Whitney test. *p<0.05.

As a marker of bacterial burden, we quantified the fluorescence intensity of a reporter-strain of *M*. *marinum*, which enabled us to measure the progression of infection in real-time for each embryo. Whilst we observed a significantly reduced bacterial burden in the Bac5-treated condition, this was only apparent at 72 hpi, and the overall trend was still increased bacterial burden over time, suggesting the peptide treatment may slow, but not prevent colonisation by *M*. *marinum* (**Fig 5**).

To investigate whether Bac5 could affect bacterial burden of an established infection, we allowed infection to proceed unchallenged until administering Bac5 at 48 hpi, with a repeat treatment at 96 hpi. However, in this model there was also no protective benefit of the AMP treatment, either in reducing bacterial burden or in embryo survival (**S5 Fig**).

## Discussion

Mycobacteria are able to redirect the host immune response from one that is able to clear the infection (Th1) towards one that supports persistence (Th2) that can last for many years [44]. Dysregulation of the immune response can also result in excessive and damaging immunopathology [31, 45]; AMP may offer a solution for restoring an effective host response. In the present work, we sought to characterise the immunomodulatory properties of the proline-rich bovine AMP, Bac5. In particular, we wanted to examine its *in vivo* potential using the embryonic zebrafish model in the context of early infection with virulent *M*. *marinum*, a close relative of *M*. *tuberculosis*.

### *In vitro* activity of Bac5

Bac5 was found to broadly restrict metabolic activity across a number of different bacterial species. However, its killing activity was more species-selective, with *Y*. *pseudotuberculosis* and *M*. *bovis* BCG demonstrating greater resistance to the peptide than other species, although they were still sensitive to higher concentrations. These findings fit with observations that Bac5 can fuse with bacterial membranes [18] and with reports that Bac2 is active against Gram-negative bacilli [15]. A suggested target for Bac5 is through the ABC transporter, SbmA, which facilitates uptake of the peptide. While SbmA is conserved amongst Gram-negative bacteria, it is not among Gram-positive species (24). We are the first to report Bac5 microbicidal activity against mycobacterial species; interestingly, a BLAST comparison revealed that *Mycobacterium spp*. possess an orthologous ABC transporter, to SbmA, but with only 24% identity. The mechanism of uptake and killing by Bac5 therefore remains to be proven.

We found that Bac5 was able to significantly upregulate the transcription of both *IL-1β* and *TNF-α* in mature THP-1 cells, indicating that it can activate macrophage-like cells *in vitro*. The induction of *IL-1β* by Bac5 was of interest since it is reported to be an important mediator of host resistance to MTB infection [46–48]. *IL-1* gene knockout studies have shown its importance in reducing bacterial burden and improving survival in mice [49–51], while mice that are deficient in IL-1 receptor demonstrate greater necrosis in response to MTB infection [50, 52].

Since we detected concomitant *IL-1β* and *TNF-α* transcriptional activity, we could not determine whether Bac5 directly upregulated *TNF-α* since it has been reported that IL-1β can itself induce *TNF-α* [53–55]. Interestingly, our findings contrast with the reported inability of Bac5 to upregulate *TNF-α* in bovine epithelial cells [26], suggesting this may be a cell-type specific effect. The ability of Bac5 to activate THP-1 cells optimally required co-stimulation with *M*. *marinum*. This is analogous to the classical activation pathway of macrophages which requires two signals: a pathogen-associated molecular pattern (PAMP) and a pro-inflammatory cytokine such as IFN-γ [56, 57]. It contrasts with human LL37, which could kill *M*. *marinum*, but not induce *TNF-α* in our hands.

While TNF-α is important in the host response to mycobacterial infection, it can also promote tissue damage if unregulated [58–60]. For this reason, we were particularly interested to examine co-induction of *MMP-9*. MMP-9 is a metalloprotease that has been associated with macrophage recruitment and wound healing, and the formation of well-defined and stable granuloma in both murine and zebrafish models of mycobacterial infection [61, 62]. We found that epinecidin induced MMP-9 in synergy with *M*. *marinum* in THP-1 cells. With Bac5, the response was variable, with only about half the replicates showing strong induction of *MMP-9* in response to Bac5 treatment. In A549 cells endogenously expressing Bac5, however, TNF-α stimulation induced significantly higher amounts of MMP-9 protein. It is interesting that Bac5 is able to both induce *TNF-α* transcription in one cell type, and enhance sensitivity to it in another, and suggests it can potentially act as a signal mediator in support of cytokine function. In constructing stable cell lines expressing AMP, we demonstrate a proof of principle that these peptides can be produced more efficiently than existing synthetic methods such as those using solid-phase chemistry [63], or using bacterial or yeast systems that are complicated by auto-toxicity issues.

### *In vivo* activity of Bac5

We identified macrophage recruitment *in vivo* in the presence of Bac5 that was sustained for 24 hours, independent of *M*. *marinum* infection. However, when bacteria and peptide were co-injected, Bac5 promoted no additional macrophage recruitment above the level induced by *M*. *marinum* alone. The ability of Bac5 to recruit macrophages independently may support an early immune response when the peptide is located away from the immediate proximity of a nascent infection. From a wider perspective, the ability of Bac5 to recruit macrophages highlights a potential application as a peptide-based vaccine adjuvant, considering that *M*. *bovis* BCG vaccine has been shown to promote better protection against MTB challenge when activated macrophages are present [64].

We also observed a chemotactic effect of Bac5 on neutrophils at six hpi in synergy with *M*. *marinum*, although the effect was not sustained over longer time periods. The lack of significant *cxcl-c1c* transcriptional activity *in vivo* suggested that Bac5 did not recruit neutrophils via induction of this chemokine.

Treatment with Bac5 resulted in significantly reduced bacterial burden at 72 hpi, potentially due to the observed early enhanced recruitment of macrophages and neutrophils. While neutrophils reportedly lack the ability to efficiently phagocytose and kill free mycobacteria [65], there is evidence that they can support killing by macrophages, as well as engulf and destroy mycobacteria-infected macrophages [66].

Still, we only observed a reduced rate of bacterial infection, not control of or killing of mycobacteria, and we suggest this may be due to an overall lack of pro-inflammatory cytokine induction in the *in vivo* model, exacerbated by a lack of adaptive immunity. This is likely to significantly affect the ability of the host to resist infection, and may limit the contribution of Bac5.

While TNF-α produced by TLR-stimulated macrophages can activate macrophages, it optimally requires co-stimulation with IFN-γ [67, 68], of which T-cells are a major source. CD4^+^ Th1 T-cell-produced IFN-γ and TNF-α can activate bystander resting macrophages and prevent their infection by MTB [44, 69]. Non-activated macrophages, by contrast, are permissive to mycobacterial infection [70, 71]. Clay *et al* have shown that macrophages are sufficient to control bacillary burden of *M*. *marinum* in the embryonic zebrafish model without the contribution of adaptive immunity [72]; however in that study, a high level of tnfa was found, suggesting the macrophages were activated. There are conflicting reports of the ability of *M*. *marinum* to induce *tnf* in zebrafish embryos. Some have described it to be a potent inducer of *tnfa* [72, 73], whereas others have reported only weak or delayed induction [74, 75]. In our hands, *M*. *marinum* was able to upregulate *il-1β*, but it was only detected in significant amounts after 96 hours. Bac5, a potent inducer of *il-1β in vitro*, failed to upregulate it *in vivo*. A possible explanation for the lack of either *tnfa*, *il-1β* or *mmp-9* transcriptional activity observed *in vivo* may be the presence of TGF-β in the developing embryo [76], which has been described to inhibit IL-1β, TNF-α [77] and MMP-9 production [78].

## Conclusions

AMP are a key component of the innate immune system, we therefore sought to study the function of Bac5 purely in this context, modelling early infection of a host with a natural pathogen. We furthermore utilised *in vitro* systems to study the effects of Bac5 in a reductionist approach. By definition, these models do not fully replicate the role of Bac5 in the bovine system. Determining the physiological concentration of AMPs is notoriously difficult, but some data is available. For context, the concentration of LL37 stored in human neutrophils and found in plasma and adult airway fluid is in the range of 0.6–2 μg mL^-1^[79]. In our present work, we injected 10 ng Bac5 per embryo; the chemotactic effects we observed therefore represent a potent function of Bac5. Additionally, in a physiological setting, the peptide itself would be continually replenished by neutrophil degranulation, whereas our design represents a single ‘pulse-chase’ kinetic. Since the *in vivo* half-life of some AMPs can be just a few minutes [3], it would be interesting to examine the impact of Bac5 when constitutively expressed *in vivo* rather than using exogenous synthetic peptide.

In conclusion, we have demonstrated direct bactericidal activity of the proline-rich bovine innate immune peptide, Bac5. For the first time, we show that Bac5 is able to variably kill some mycobacterial species, and it supports macrophage activation *in vitro* in synergy with *M*. *marinum*. We also show that it is able to induce recruitment of macrophages, and that it can enhance recruitment of neutrophils in the presence of *M*. *marinum*. A single dose of the peptide was able to slow but not prevent infection of zebrafish embryos with *M*. *marinum*; a lack of inflammatory cytokine induction *in vivo* may limit the potency of Bac5, particularly in the absence of adaptive immunity. These findings point to Bac5 being a potentiator of the innate immune response in response to infection beyond its recognised ability to kill Gram-negative bacteria.

## Materials and methods

### Stable expression of Bac5 in A549 human epithelial cells

To create stable A549 human epithelial cell lines expressing the active form of Bac5, we began by synthesising the double-stranded DNA encoding Bac5 using overlap extension PCR with primers for the entire coding sequence with Kozak sequence inserted at the 5’ end and using an A-tailing polymerase. The sequence was directly cloned into pcDNA 3.3 (Invitrogen, UK) and used to transform TOP10 *E*. *coli* and screened by ampicillin resistance and colony PCR. Positive clones were confirmed by sequencing of plasmid minipreps (Qiagen Spin Miniprep). Since the expression of the antimicrobial peptide is under the control of a CMV promoter, it is not expressed during *E*. *coli* replication and so there is no auto-toxicity. Purified pcDNA3.3_Bac5 plasmid were linearised and introduced into human A549 cell lines (ATCC-LGC) using Lipofectamine^TM^ (Thermo Fisher Scientific) transfection reagent, and after 24 hours culture at 37 ^o^C with 5% CO_2_ in DMEM Glutamax (Gibco) with 10% foetal calf serum, 100 μg ml^-1^ gentamycin (Sigma Aldrich) was added to the cell culture to select for chromosomally integration and expression of the vector. Expression of Bac5 was confirmed by RNA extraction from the cell lines followed by cDNA synthesis and PCR using Bac5-specific primers.

### Synthetic peptides

All synthetic peptides were manufactured by Biomatik. All AMPs were resuspended in nuclease-free water to a stock concentration of 10 mg/ml. Sequences were as follows: Bac5 **=** RFRPPIRRPPIRPPFYPPFRPPIRPPIFPPIRPPFRPPLGPF; Epinecidin = GFIFHIIKGLFHAGKMIHGLV; LL37 (Cathelicidin) = LLGDFFRKSKEKIGKEFKRIVQRIKDFLRNLVPRTES.

### Bacterial strains and maintenance

*E*. *coli* (strain HB101) was maintained at 37°C in Luria-Bertani (LB) media or on LB agar plates. *M*. *marinum* (strain M, ATCC BAA-535) was maintained at 28.5°C in Middlebrook 7H9 media (BD), or on Middlebrook 7H11 or 7H10 plates (BD) supplemented with 10% Middlebrook Oleic Acid Albumin Dextrose Complex (OADC) media (BD and Stratech). All liquid cultures were incubated with shaking at 150 rotations per minute (rpm) and liquid cultures of *M*. *marinum* also contained 0.05% Tween-80. Where appropriate, growth media were supplemented with 25 μg/ml kanamycin and/or 50 μg/ml hygromycin. Where stated, *M*. *marinum* (strain M, ATCC BAA-535) containing pMSP12:DsRed2[80] or pGFPHyg2[81] was used and maintained as above.

### *In vitro* activity of endogenous-expressed Bac5

To assess the activity of endogenously produced Bac5 peptide, cell culture supernatant was collected from either A549 cells or Bac5_A549 cells. Alternatively, adherent cells were washed three times with sterile PBS before harvesting using Accutase (Thermo Fisher Scientific) and mechanical lysis using a sterile douncer. Protein concentration was normalised using modified Bradford Assay, and equivalent amounts of either cell lysate or cell culture supernatant added 1:1 with lysogeny broth containing 1 x 10^5^ CFU bacteria, either *M*. *smegmatis*, *M*. *bovis* BCG, *Burkholderia*. *thailandensis*, *Yersinia pseudotuberculosis* IP32953 or *E*. *coli*. 96-well plates were incubated at 37°C for 24 hours before the addition of Alamar Blue reagent (Thermo Fisher Scientific) for 1 hour and measurement of optical density at 590 and 620 nm. Metabolic activity was calculated and normalised against the bacteria-free control.

To assess the effect of constitutive Bac5 expression on the responsiveness of the A549 cells to mycobacterial PAMP or cytokine stimulation, we incubated 1x 10^5^ cells in a 96 well plate with different MOI of either *M*. *smegmatis*, *M*. *bovis* BCG, or TNF-α. After 24 hours incubation at 37 ^o^C, cell culture supernatant was harvested and centrifuged to pellet extracellular bacteria. Supernatant used measured for metalloprotease (MMP-9) and CXCL8 content by enzyme-linked immunosorbent assay (ELISA) according to manufacturer’s instructions (both Thermo Fisher Scientific).

### *In vitro* activity of synthetic AMPs assay

*M*. *marinum* starter cultures were used at OD_600_ 1.0 and *E*. *coli* starter cultures at OD_600_ 0.5 (ensuring the bacteria were in logarithmic phase) before dilution in a solution of 1:4 (v:v) RPMI:water to a concentration of 1.5x10^6^ CFU per ml. 100 μl of bacterial solution (1.5x10^5^ CFU /well) was added to each well with 10 μl of appropriate 10X working AMP stock (prepared fresh in 1:4 (v:v, RPMI:water) and the 96-well plate incubated for 24 hours at 28.5 ^o^C. For *M*. *marinum* experiments, serial dilutions in 1X PBS were plated on 7H10 agar for each well and CFU counts taken after 5 days of incubation at 28.5 ^o^C. For *E*. *coli* experiments, serial dilutions in 1X PBS were plated on LB agar for each well and CFU counts taken after overnight incubation at 28.5 ^o^C. Three biological replicates per experiment were performed for each AMP concentration.

### THP-1 cells maintenance

The THP-1 acute monocytic leukaemia cell line (ATCC TIB-202) was maintained in RPMI 1640 Medium, GlutaMAX supplement (Gibco) containing 10% FBS (heat-inactivated, Sigma-Aldrich), 50 units/ml penicillin and 50 μg/ml streptomycin (penicillin/streptomycin, Sigma-Aldrich). Cells were maintained at a density of 2x10^5^ - 1x10^6^ cells at 37°C 5% CO_2_ in a humidified chamber. All procedures were carried out using cells between passages 9 and 12.

### THP-1 cell assays with AMPs

THP-1 cells were plated at 5x10^5^ cells/well to 96-well plates in RPMI 1640 Medium, GlutaMAX supplement containing 10% FBS, 50 units/ml penicillin and 50 μg/ml streptomycin. Following plating, cells were incubated for 24 hours at 37°C 5% CO_2_ with or without activation with 20 ng/ml PMA (phorbol 12-myristate 13-acetate, Sigma-Aldrich) as stated in figure legends. Cell media was changed to RPMI 1640 Medium, GlutaMAX supplement containing 10% FBS only, and all cells incubated for a further 24 hours without PMA or antibiotics. Meanwhile, *M*. *marinum* was cultured to an OD_600_ of 1.0, bacteria harvested by centrifugation, and bacteria resuspended in RPMI 1640 Medium, GlutaMAX supplement containing 10% FBS only. Following 24 hours rest without PMA, 10 μl of bacterial suspension or 10 μl media only was added to each well. Infection with *M*. *marinum* at MOI 1:1 was allowed to proceed for 3 hours at 33°C 5% CO_2_, cells were washed 3x with RPMI 1640 Medium, GlutaMAX supplement (no additives), and cell media was replaced with RPMI 1640 Medium, GlutaMAX supplement (no additives) containing no AMP or 15 ng/μl of the AMP indicated. Cells were then incubated for the stated times at 33°C 5% CO_2_.

qRT-PCR sampling was performed by adding 100 μl lysis buffer (MagMAX-96 Total RNA Isolation Kit) per sample, and proceeding with sample processing according to the manufacturer’s recommendations. Pools of two wells were used for each sample by transferring the 100 μl lysis buffer from the first well to the second before processing the sample.

### Zebrafish lines and maintenance

Zebrafish lines used in this study were *Tg(mpeg-1*:*mCherry)gl23*[82], *casper Tg(mpx*:*GFP)i114*[83–85] and *casper Tg(Lyz*:*GFP)nz117*[86]. All zebrafish embryos in this study were under 6dpf and larvae were maintained at 28.5°C in 0.5X E2[87], including infected zebrafish. Wildtype zebrafish were purchased from the Zebrafish International Resource Center (Eugene, OR). Survival was monitored daily and dead zebrafish embryos and larvae removed. Where required for injection or imaging procedures, zebrafish embryos and larvae were anaesthetised in E2 containing 4.2% MS-222 (3-aminobenzoic acid ethyl ester, Sigma-Aldrich), followed by recovery in 0.5X E2 media containing no anaesthetic. Where euthanasia was performed, zebrafish embryos and larvae were terminally anaesthetised in 0.5X E2 containing 15% MS-222 and death verified after overnight incubation. To prevent melanisation and aid imaging, *Tg(mpeg-1*:*mCherry)* zebrafish were maintained from 24hpf in 0.5X E2 containing 30 mg/L PTU (1-phenyl-2-thiourea, Sigma-Aldrich).

### Zebrafish injections

Embryos were dechorionated manually prior to injections using fine forceps. Hindbrain ventricle (HBV) injections were performed at 2 days post-fertilisation (dpf, 50–54 hpf) with an IM 300 microinjector with injection volumes of approximately 1 nl. Embryos were injected with one of four possible injection solutions during this study, as specified in figure legends: 1X PBS (mock); 1X PBS containing *M*. *marinum*; 10 mg/ml BAC; or 10 mg/ml BAC containing *M*. *marinum*. The solution of 1X PBS was chosen as an osmotically-balanced non-inflammatory control (mock) injection. All injection solutions contained phenol red dye (Sigma-Aldrich) at a final concentration of 0.0005%.

For zebrafish injections, *M*. *marinum* expressing DsRed2[80] or GFPmut3[81] was grown to mid-logarithmic phase (OD_600_ 1.0). Growth curves were initially performed for each strain to determine the appropriate dilutions; bacterial cultures were concentrated 5-fold for injections. Cells were harvested by centrifugation, washed 3X in 1X PBS, and resuspended at the appropriate dilution for injections in 1X PBS for *M*. *marinum* only injections. Co-injection solutions were first resuspended at the appropriate dilution in 100 μl 1X PBS to provide the Mock/*M*. *marinum* injection solution, with 10 μl of this preparation then being harvested by centrifugation and resuspended in 10 μl of 10 mg/ml Bac5 to provide the Bac5/*M*. *marinum* injection solution. Injection doses were verified by injecting bacterial solutions directly into a 10 μl drop of sterile 1X PBS and plating the drop to 7H10 for CFU counting after 5 days of incubation. Three 1X PBS drop injections were plated for each experiment.

Zebrafish were sampled for qRT-PCR following injections by euthanizing the zebrafish, homogenising the samples using a pestle (Kimble-Chase) in 100 μl lysis buffer (MagMAX-96 Total RNA Isolation Kit), and proceeding with sample processing using the kit according to manufacturer’s recommendations. qRT-PCR was performed for pools of 3 whole zebrafish embryos per sample at 6 hpi and 24 hpi, or individual zebrafish larvae samples at 96 hpi.

### Zebrafish microscopy and image quantification

Live zebrafish embryos and larvae were imaged using a Leica M205 FA fluorescence stereomicroscope. To enable quantification of infection progression parameters from images of zebrafish, transgenic zebrafish lines expressing fluorescently labelled immune cells and fluorescently-labelled *M*. *marinum* strains were used. Z-stack images of the zebrafish HBV region were acquired in the dorsal view with the z-stack range capturing the entire head region from the dorsal to ventral sides. All images were acquired for infected and uninfected zebrafish using standardised settings for each infection parameter to be quantified such that repeat experiments could be compared and/or data pooled. All zebrafish were manually positioned in the dorsal orientation to ensure the accuracy of parameter quantification from images. Data for zebrafish which died or suffered from pericardial and/or yolk sac oedema during the course of experiments was excluded from the analyses presented for all time points. All quantifications and brightness and contrast adjustments for images presented in this study were performed in Icy (open-source image analysis software, http://icy.bioimageanalysis.org/).

To permit the quantification of HBV region leukocyte recruitment and bacterial burden following injections, images were first processed. The in-focus z-slice was extracted from z-stacks for analysis. The selected z-slice was defined as the depth of slice from the top of the head in which bacterial fluorescence was seen in the majority of infected zebrafish groups; slices at that depth were then extracted and processed for all zebrafish in the experiment. To rapidly and reproducibly define a standardised HBV region for analysis, selected z-slice images were cropped at the otic vesicle, such that quantification was performed for the zebrafish head region from the tip to the otic vesicle—referred to as the “HBV region” throughout this study. Cropping facilitates the removal of the yolk sac and lymph nodes from the regions to be processed, eliminating issues with autofluorescence and large collections of immune cells in images, respectively.

The processed z-slice was analysed using both an Icy FPC protocol to determine the bacterial burden, and the Icy Spot Detector plugin to determine leukocyte recruitment to the HBV region. The FPC protocol is based upon the Icy Thresholded Pixel Density plugin (developed by Fabrice de Chaumont, with the FPC protocol provided as “Pixel Density (batch mode) protocol” on the Icy website) and integrates the number of pixels in each image of infected zebrafish with values above a background threshold, as determined by matched images of uninfected animals. The background threshold intensity is defined as the value for the highest intensity pixel in the uninfected control images. The Icy Spot Detector plugin [88] detects the number of pixel clusters of user-defined scale and sensitivity within an image, and was used to detect the number of fluorescently-labelled cells present in an image. In addition to quantification using the Spot Detector plugin, the Icy FPC protocol was also used to quantify neutrophil recruitment to the HBV region in co-injection experiments (Fig 4). This was due to imperfect resolution of neutrophil cell clusters at the 72 hpi time point by the Icy Spot Detector plugin; caused by the use of standardised image acquisition settings throughout these experiments from 6 to 72 hpi to permit comparison of the results at different time points.

To enable the separation of zebrafish into equal groups of infection burden and distribution for sequential injection experiments, single images of the HBV region were acquired in the dorsal view with the infecting bacteria in-focus at 24 hpi. These images were cropped at the otic vesicle as described above to provide the HBV region for analysis. The processed images were analysed using the Icy FPC protocol as above to determine the bacterial burden, zebrafish were separated into four groups of equal infection burden and any surplus infected zebrafish were discarded at this point. The four groups of zebrafish were then treated with sequential injections of Mock or Bac5 as described in figure legends.

### qRT-PCR

Following RNA extraction from samples using the MagMAX-96 Total RNA Isolation Kit (Ambion), RNA quantity and quality was determined using a NanoDrop 1000 (Thermo Scientific). The High-Capacity cDNA Reverse Transcription Kit (Applied Biosystems) was used according to manufacturer’s recommendations with 125 ng of total RNA sample per reaction. qRT-PCR was performed with 2% of the generated cDNA per reaction using the Taqman Fast Universal PCR Master mix, no AmpErase UNG (Applied Biosystems) and Taqman primer and probes assays (Applied Biosystems). All reactions were performed in duplicate using a 7500 Fast Real-Time PCR System (Applied Biosystems). Obtained cycle thresholds were normalised to 18S and expressed relative to one control sample for each experiment. Taqman primer and probes assays used were: 18S rRNA (4319413E), *il-1β* (Dr03114368_m1), *mmp-9* (Dr03139882_m1), *TNF-α* (Dr03126850_m1), *cxcl-c1c* (Dr03436643_m1), and *ifn-phi1* (Dr03100938_m1).

### Statistical analysis

All statistical analyses were carried out using GraphPad Prism 4.0 software (GraphPad Software, La Jolla California USA, www.graphpad.com). The D’Agostino-Pearson omnibus test was used to confirm a normal distribution of data (parametric data). To compare two groups, unpaired two-tailed T-tests or Mann-Whitney tests were used for parametric or non-parametric datasets, respectively. To compare more than two groups, one-way ANOVA followed by Bonferroni’s multiple comparison test or Kruskal-Wallis test followed by Dunn’s multiple comparison test were used for parametric or non-parametric data, respectively. P values of less than 0.05 were deemed statistically significant with *** p<0.001, ** p<0.01 and * p<0.05.

## Supporting information

S1 Fig Single injection of Bac5 to the HBV does not affect *il-1β, mmp-9, tnfa* or *cxcl-c1c* transcription in uninfected or *M. marinum-*infected zebrafish. Two dpf *Tg(mpeg-1*:*mCherry)* zebrafish embryos were injected with Mock, 10 ng Bac5, Mock/*M*. *marinum* (Mmar) (approx. 190 CFU) or Bac5/Mmar. Fold changes of *il-1β*, *mmp-9*, *cxcl-c1c* and *tnfa* mRNA levels were assessed by qRT-PCR, normalised to 18S and expressed relative to one control uninjected sample. Samples were taken at 6 hpi (**A**), 24 hpi (**B**) and 96 hpi (**C**). Each data point in (A-B) represents a pool of three fish (n = 33 for uninjected and Bac5/Mmar injected groups at 24 hpi, and n = 36 for all other groups at 6 hpi and 24 hpi), each data point in (C) represents an individual fish (n = 15). Data were pooled from three independent experiments. Error bars represent S.E.M. Mann-Whitney test for 96 hpi *tnfa* data and Kruskal-Wallis with Dunn’s post-test for all other data. ***p<0.001. Statistical significance between *M*. *marinum-*infected and uninfected groups is displayed relative to the Mock control group only for clarity. The uninjected control group is shown for reference only; statistical significance to this group is not shown for clarity.
(TIF)

S2 Fig Delayed injection of Bac5 to the HBV of *M. marinum-*infected zebrafish at 72hpi or 90hpi does not affect cytokine transcription. Two days post-fertilisation *Tg(mpeg-1*:*mCherry)* zebrafish embryos were injected into their HBV with approximately 180 CFU *M*. *marinum* expressing GFP. Zebrafish embryos were live-imaged using a fluorescence stereomicroscope at 24 hpi, individual bacterial burdens calculated using the Icy FPC protocol, and embryos were separated into four groups of equal infection burden and distribution. Zebrafish were then injected with either Mock or 10 ng BAC at 72 hpi or 90 hpi. Fold changes of *il-1β*, *mmp-9*, *tnfa* and *cxcl-c1c* mRNA levels were assessed at 96 hpi by qRT-PCR, normalised to 18S and expressed relative to one control sample. Data pooled from two independent experiments is shown (n = 19 for 90 hpi Mock injection group and n = 20 for all other groups). Each data point represents an individual fish. Error bars represent S.E.M. Kruskal-Wallis with Dunn’s post-test. *p<0.05, **p,0.01.
(TIF)

S3 Fig Endogenously expressed Bac5 confers antibacterial activity and enhances cytokine and chemokine response to infection. A549 cells and A549 cell lines stably expressing *Bac5* were tested for ability to restrict bacterial metabolic activity. Cell lysate (**A**) or culture supernatant (**B**) were incubated neat with different species of bacteria (MOI of 1) and metabolic activity was assessed after 24 h by Alamar Blue assay. To asses chemokine and cytokine responses, cell lines were seeded at 1 x 10^5^ in 96-well plate and challenged with different MOI of bacteria, or 10–100 ng/ml TNFα over 24 hrs. Cell culture supernatant was used to perform either MMP-9 (**C**) or CXCL8 (**D**) ELISA and normalised against the untreated negative control. * p<0.01 Student’s T-test.
(TIF)

S4 Fig Delayed injection of Bac5 does not affect neutrophil recruitment in infected embryos. Two days post-fertilisation *casper Tg(LysC*:*GFP)* zebrafish embryo groups were injected into their HBV with approximately 220 CFU *M*. *marinum* expressing DsRed2. Zebrafish embryos were live-imaged using a fluorescence stereomicroscope at 24 hpi, individual bacterial burdens calculated using the Icy FPC protocol, and embryos were separated into four groups of equal infection burden and distribution. Two groups were treated with Mock or 10 ng Bac5 at 48 hpi and two groups were treated with repeated doses of Mock or 10 ng Bac5 at 48 hpi and 96 hpi. Zebrafish embryos were live-imaged using a fluorescence stereomicroscope and z-stack images of the HBV region acquired at 72 hpi and 120 hpi. **(A-F)** Representative fluorescence images of fish from the same single experiment are shown at 72 hpi (A-B) and 120 hpi (C-F). Images of the neutrophil and bacterial fluorescence channels are shown separated by white dashed line. Scale bar 100 μm. **(G-H)** Neutrophils in the HBV region were quantified from fluorescence images of zebrafish embryos using Icy Spot Detector plugin at 72 hpi (**G**) and 120 hpi (**H**). Sample size (n): 111, 109 (**G**) and 47, 43, 45, 41 (**H**). Data pooled from three independent experiments is shown. Error bars represent S.E.M. Unpaired t-test (**G**), one-way ANOVA with Bonferroni’s post-test (**H**) showed no significant differences.
(TIF)

S5 Fig Delayed injection of Bac5 does not affect bacterial burden or survival of infected embryos. Two days post-fertilisation *casper Tg(LysC*:*GFP)* zebrafish embryos were injected into their HBV with approximately 220 CFU *M*. *marinum* expressing DsRed2. Zebrafish embryos were live-imaged using a fluorescence stereomicroscope at 24 hpi, individual bacterial burdens calculated using the Icy FPC protocol, and embryos were separated into four groups of equal infection burden and distribution. Two groups were treated with mock or 10 ng Bac5 at 48 hpi and two groups were treated with repeated doses of mock or 10 ng Bac5 at 48 hpi and 96 hpi. Zebrafish embryos were live-imaged using a fluorescence stereomicroscope and z-stack images of the HBV region acquired at 72 hpi and 120 hpi (**A**). Survival of all zebrafish embryo treatment groups was recorded, including dosed with single injection of Bac5 at 48 hpi and with second injection at 96 hpi (**B**). Data pooled from three independent experiments is shown. Error bars represent S.E.M. Sample size, 111 and 109 (72 hpi) and 47, 43, 45, 41 (120 hpi). Kruskal-Wallis with Dunn’s post-test (**A**) and survival analysis (**B**) showed no significant differences.
(TIF)
