# Supplementary figures and images for: In vitro and in vivo properties of the bovine antimicrobial peptide, Bactenecin 5
Source: PLoS One. 2019 Jan 9;14(1):e0210508. doi: 10.1371/journal.pone.0210508 (PMC6326515; doi:10.1371/journal.pone.0210508)

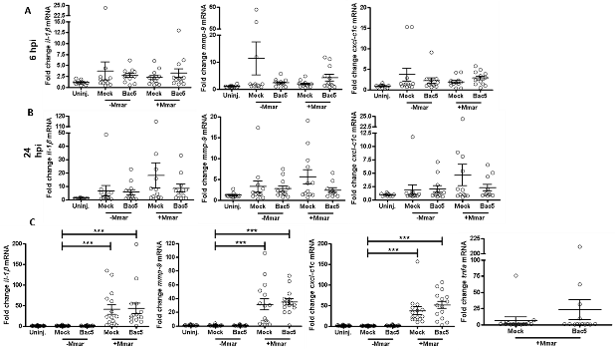

Supplement: S1 Fig — Two dpf Tg(mpeg-1:mCherry) zebrafish embryos were injected with Mock, 10 ng Bac5, Mock/M. marinum (Mmar) (approx. 190 CFU) or Bac5/Mmar. Fold changes of il-1β, mmp-9, cxcl-c1c and tnfa mRNA levels were assessed by qRT-PCR, normalised to 18S and expressed relative to one control uninjected sample. Samples were taken at 6 hpi (A), 24 hpi (B) and 96 hpi (C). Each data point in (A-B) represents a pool of three fish (n = 33 for uninjected and Bac5/Mmar injected groups at 24 hpi, and n = 36 for all other groups at 6 hpi and 24 hpi), each data point in (C) represents an individual fish (n = 15). Data were pooled from three independent experiments. Error bars represent S.E.M. Mann-Whitney test for 96 hpi tnfa data and Kruskal-Wallis with Dunn’s post-test for all other data. ***p<0.001. Statistical significance between M. marinum-infected and uninfected groups is displayed relative to the Mock control group only for clarity. The uninjected control group is shown for reference only; statistical significance to this group is not shown for clarity. (TIF) [file pone.0210508.s001.tif]

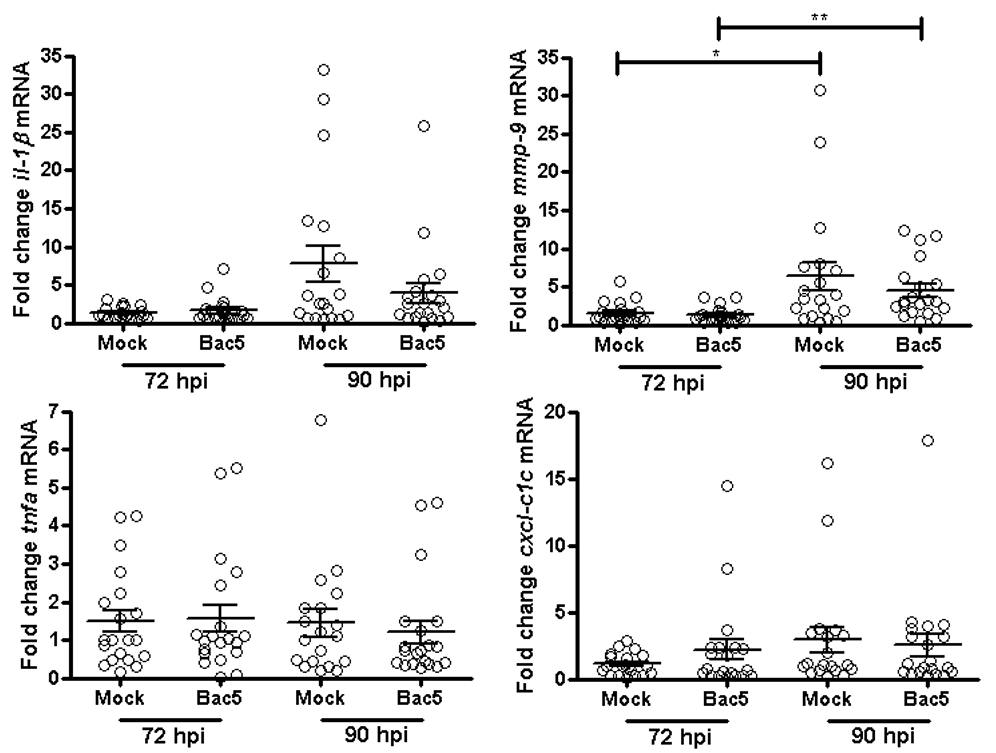

Supplement: S2 Fig — Two days post-fertilisation Tg(mpeg-1:mCherry) zebrafish embryos were injected into their HBV with approximately 180 CFU M. marinum expressing GFP. Zebrafish embryos were live-imaged using a fluorescence stereomicroscope at 24 hpi, individual bacterial burdens calculated using the Icy FPC protocol, and embryos were separated into four groups of equal infection burden and distribution. Zebrafish were then injected with either Mock or 10 ng BAC at 72 hpi or 90 hpi. Fold changes of il-1β, mmp-9, tnfa and cxcl-c1c mRNA levels were assessed at 96 hpi by qRT-PCR, normalised to 18S and expressed relative to one control sample. Data pooled from two independent experiments is shown (n = 19 for 90 hpi Mock injection group and n = 20 for all other groups). Each data point represents an individual fish. Error bars represent S.E.M. Kruskal-Wallis with Dunn’s post-test. *p<0.05, **p,0.01. (TIF) [file pone.0210508.s002.tif]

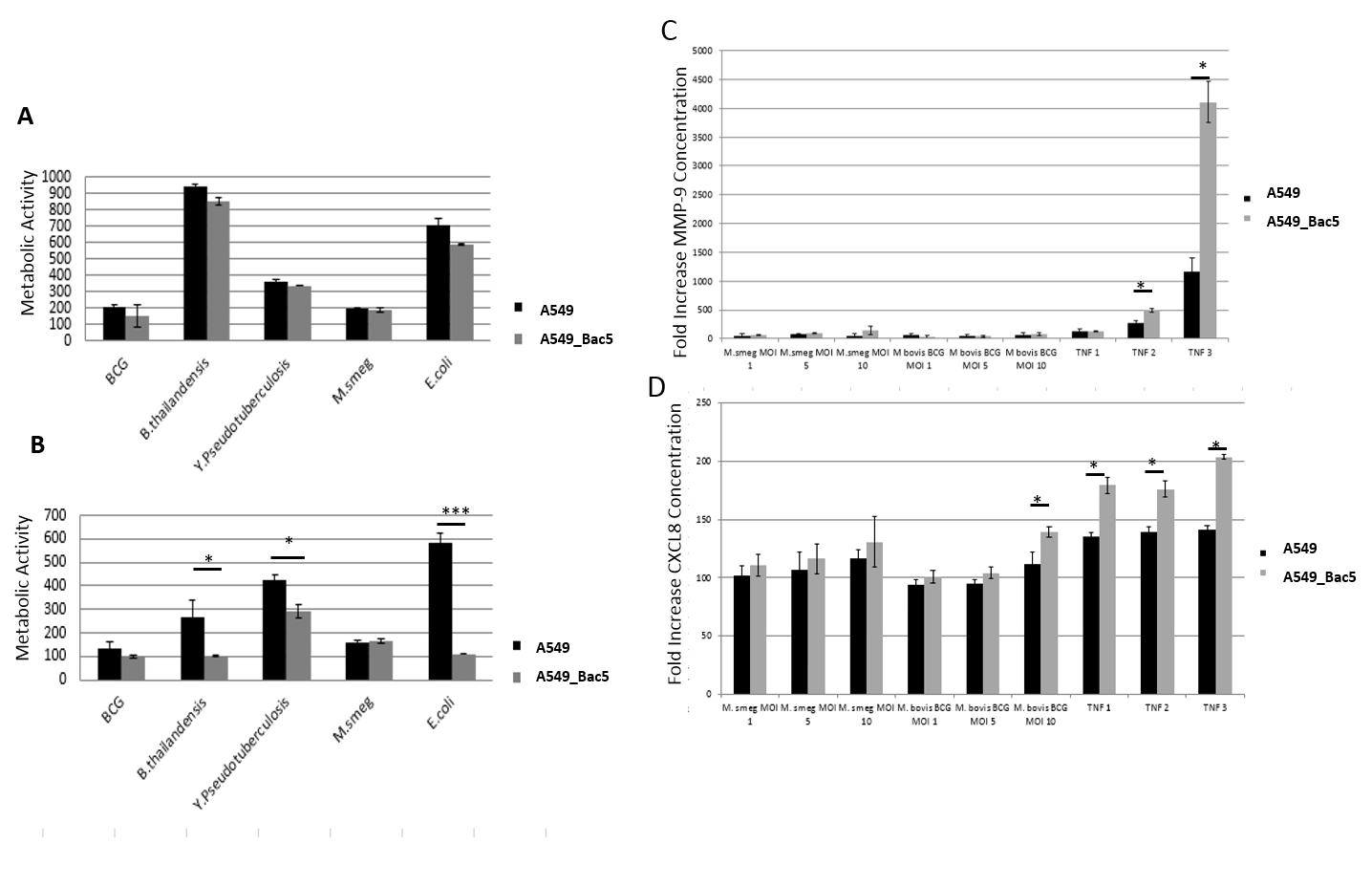

Supplement: S3 Fig — A549 cells and A549 cell lines stably expressing Bac5 were tested for ability to restrict bacterial metabolic activity. Cell lysate (A) or culture supernatant (B) were incubated neat with different species of bacteria (MOI of 1) and metabolic activity was assessed after 24 h by Alamar Blue assay. To asses chemokine and cytokine responses, cell lines were seeded at 1 x 10^5^ in 96-well plate and challenged with different MOI of bacteria, or 10–100 ng/ml TNFα over 24 hrs. Cell culture supernatant was used to perform either MMP-9 (C) or CXCL8 (D) ELISA and normalised against the untreated negative control. * p<0.01 Student’s T-test. (TIF) [file pone.0210508.s003.tif]

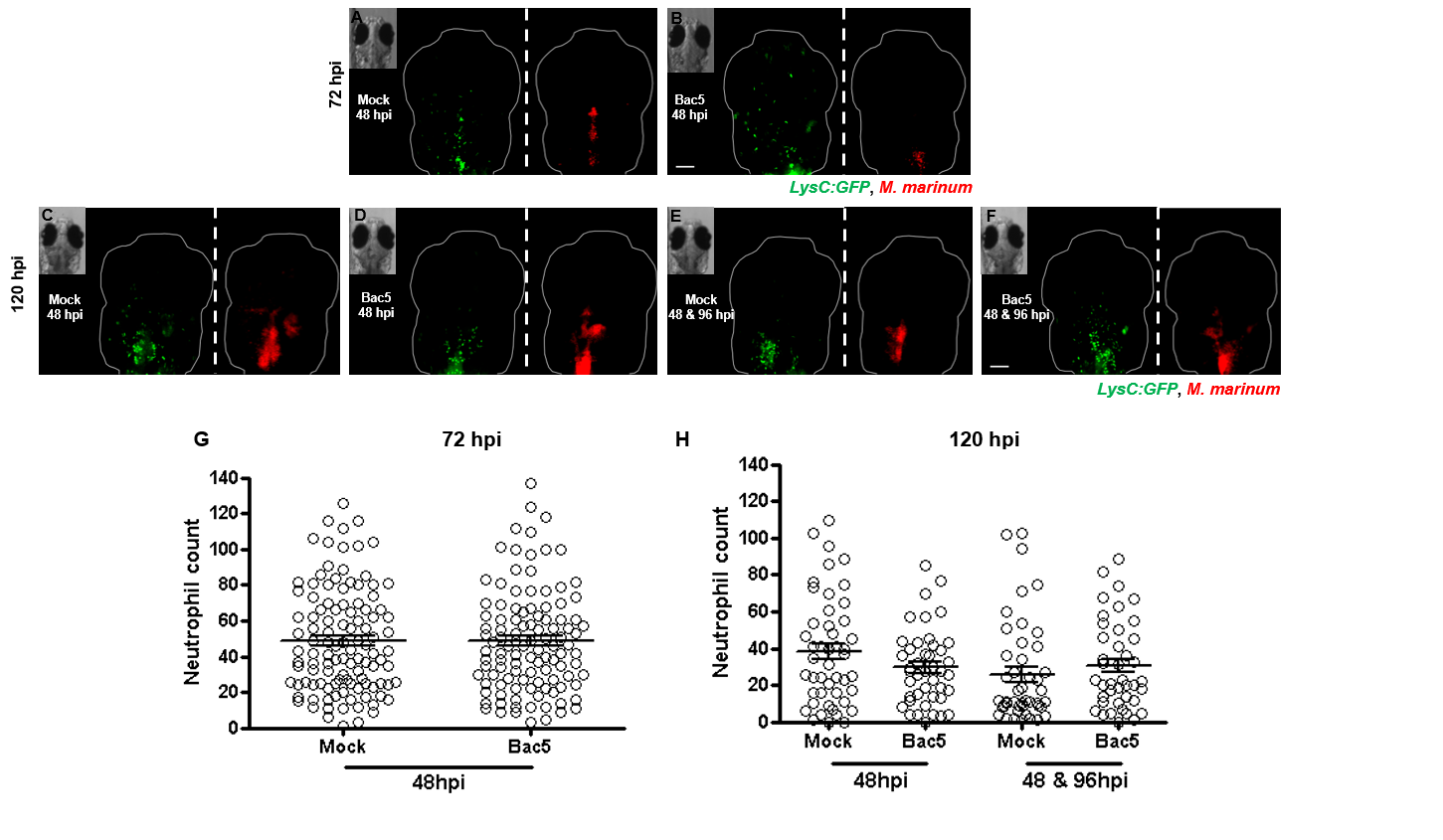

Supplement: S4 Fig — Two days post-fertilisation casper Tg(LysC:GFP) zebrafish embryo groups were injected into their HBV with approximately 220 CFU M. marinum expressing DsRed2. Zebrafish embryos were live-imaged using a fluorescence stereomicroscope at 24 hpi, individual bacterial burdens calculated using the Icy FPC protocol, and embryos were separated into four groups of equal infection burden and distribution. Two groups were treated with Mock or 10 ng Bac5 at 48 hpi and two groups were treated with repeated doses of Mock or 10 ng Bac5 at 48 hpi and 96 hpi. Zebrafish embryos were live-imaged using a fluorescence stereomicroscope and z-stack images of the HBV region acquired at 72 hpi and 120 hpi. (A-F) Representative fluorescence images of fish from the same single experiment are shown at 72 hpi (A-B) and 120 hpi (C-F). Images of the neutrophil and bacterial fluorescence channels are shown separated by white dashed line. Scale bar 100 μm. (G-H) Neutrophils in the HBV region were quantified from fluorescence images of zebrafish embryos using Icy Spot Detector plugin at 72 hpi (G) and 120 hpi (H). Sample size (n): 111, 109 (G) and 47, 43, 45, 41 (H). Data pooled from three independent experiments is shown. Error bars represent S.E.M. Unpaired t-test (G), one-way ANOVA with Bonferroni’s post-test (H) showed no significant differences. (TIF) [file pone.0210508.s004.tif]

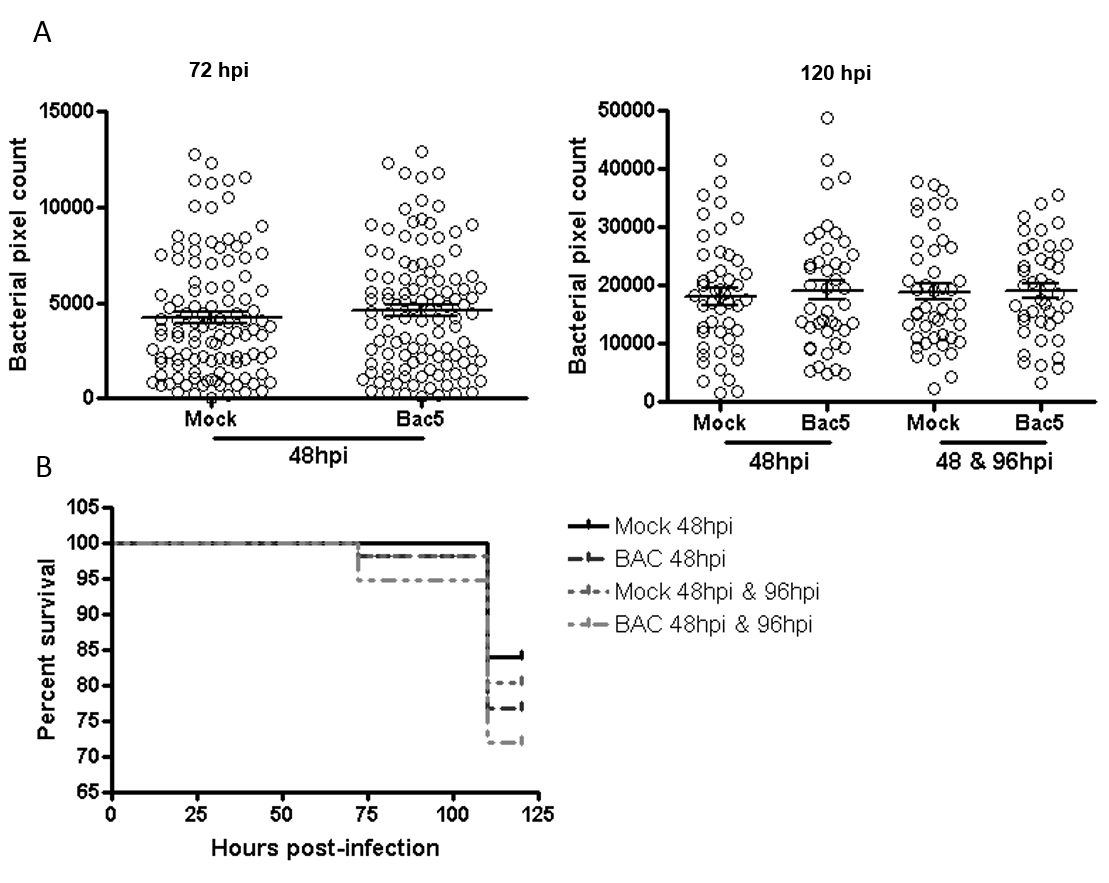

Supplement: S5 Fig — Two days post-fertilisation casper Tg(LysC:GFP) zebrafish embryos were injected into their HBV with approximately 220 CFU M. marinum expressing DsRed2. Zebrafish embryos were live-imaged using a fluorescence stereomicroscope at 24 hpi, individual bacterial burdens calculated using the Icy FPC protocol, and embryos were separated into four groups of equal infection burden and distribution. Two groups were treated with mock or 10 ng Bac5 at 48 hpi and two groups were treated with repeated doses of mock or 10 ng Bac5 at 48 hpi and 96 hpi. Zebrafish embryos were live-imaged using a fluorescence stereomicroscope and z-stack images of the HBV region acquired at 72 hpi and 120 hpi (A). Survival of all zebrafish embryo treatment groups was recorded, including dosed with single injection of Bac5 at 48 hpi and with second injection at 96 hpi (B). Data pooled from three independent experiments is shown. Error bars represent S.E.M. Sample size, 111 and 109 (72 hpi) and 47, 43, 45, 41 (120 hpi). Kruskal-Wallis with Dunn’s post-test (A) and survival analysis (B) showed no significant differences. (TIF) [file pone.0210508.s005.tif]
